# Sensitive real-time PCR detection of *Plasmodium falciparum* parasites in whole blood by erythrocyte membrane protein 1 gene amplification

**DOI:** 10.1186/s12936-019-2743-9

**Published:** 2019-04-02

**Authors:** Bryan Grabias, Edward Essuman, Isabella A. Quakyi, Sanjai Kumar

**Affiliations:** 1 Laboratory of Emerging Pathogens, Division of Emerging and Transfusion Transmitted Diseases, Center for Biologics Evaluation and Research, Food and Drug Administration, Silver Spring, MD 20993 USA; 2 Department of Biological Environmental and Occupational Health Sciences, School of Public Health, College of Health Sciences, University of Ghana, Legon, Ghana; 3 Laboratory of Emerging Pathogens, Division of Emerging and Transfusion Transmitted Diseases, Center for Biologics Evaluation and Research, Food and Drug Administration, 10903 New Hampshire Ave. Building 52-72 Rm 5304, Silver Spring, MD 20993 USA

**Keywords:** *Plasmodium falciparum* erythrocyte membrane protein 1, Parasitemia, Real-time PCR, Genomic DNA

## Abstract

**Background:**

Malaria remains a global public health problem responsible for 445,000 deaths in 2016. While microscopy remains the mainstay of malaria diagnosis, highly sensitive molecular methods for detection of low-grade sub-microscopic infections are needed for surveillance studies and identifying asymptomatic reservoirs of malaria transmission.

**Methods:**

The *Plasmodium falciparum* genome sequence was analysed to identify high copy number genes that improve *P. falciparum* parasite detection in blood by RT-PCR. *Plasmodium falciparum* erythrocyte membrane protein 1 (*Pf*EMP1)-specific primers were evaluated for *P. falciparum* detection in hospital-based microscopically positive dried blood spots and field-acquired whole blood from asymptomatic individuals from Ghana.

**Results:**

*Pf*EMP1 outperformed the *Pf*18S sequence for amplification-based *P. falciparum* detection. *Pf*EMP1 primers exhibited sevenfold higher sensitivity compared to *Pf*18S primers for parasite genomic DNA. Probit analysis established a 95% detection threshold of 9.3 parasites/mL for *Pf*EMP1 compared to 98.2 parasites/mL for *Pf*18S primers. The *Pf*EMP1 primers also demonstrated superior clinical sensitivity, identifying 100% (20/20) of dried blood spot samples and 70% (69/98) of asymptomatic individuals as positive versus 55% (11/20) and 54% (53/98), respectively, for *Pf*18S amplification.

**Conclusions:**

These results establish *Pf*EMP1 as a novel amplification target for highly sensitive detection of both acute infections from filter paper samples and submicroscopic asymptomatic low-grade infections.

## Background

In 2016, malaria caused approximately 445,000 deaths, primarily among young children in sub-Saharan Africa [1]. The apicomplexan *Plasmodium* parasites responsible for malaria-related illness are transmitted through the bite of infected mosquitoes. Of the five recognized species that infect human beings (*Plasmodium falciparum*, *Plasmodium vivax*, *Plasmodium malariae*, *Plasmodium ovale*, and *Plasmodium knowlesi*), *P. falciparum* is the most common and the most lethal. Malaria also presents a public health challenge in the USA. While there have been no cases of autochthonous transmission of malaria in the USA since 1957 [2], increasing globalization and travel to areas where malaria is endemic still results in imported malaria cases in the *Plasmodium*. In 2015 alone, there were 1186 of such travel-related malaria cases reported to the Center for Disease Control (CDC) [3] and the number of malaria hospitalizations from 2000 to 2014 far exceeded that of other common travel-related illnesses [4]. Additionally, malaria is also a major risk to the blood supply in both endemic and non-endemic countries [5–7].

The gold standard of malaria parasite detection is direct visual confirmation of intraerythrocytic parasites in a blood film. However, this method is time and labour-intensive and can only reach typical detection limits of 5000–150,000 parasites/mL of blood [8]. Several reports utilizing PCR-based methods to detect *P. falciparum* in blood have achieved far greater sensitivities than standard blood smears [9, 10]. Given the lack of knowledge on the minimum number of parasites that could be present in asymptomatic individuals and limitations in volume of blood used in detection assays, molecular methods of improved sensitivity are still needed to identify all *Plasmodium* infections in endemic areas.

Typically, most nucleic acid-based assays rely upon the detection of pathogen specific 18S ribosomal RNA (rRNA) sequences due to their universal presence, strong inter- and intra-species sequence conservation, and the relatively high abundance. While the given quantity of 18S rRNA transcripts may be higher than the number of gene copies present in an organism’s genome, RNA is typically less stable and thus requires more stringent sample handling than genomic DNA (gDNA). Depending on the strain, *P. falciparum* parasites may possess only 5–8 gene copies of the 18S ribosomal subunit [11]; therefore, the performance of assays that amplify and detect genomic sequences can be greatly enhanced with the identification of novel biomarkers that are more abundant than *Pf*18S sequences. In this report, PCR-based nucleic acid assays utilizing primers specific to the *P. falciparum* 18S ribosomal gene sequence and highly conserved regions of the abundant (approximately 60 variants [12]) *P. falciparum* var. gene EMP1 are developed and characterized. Data from analytical studies demonstrate that *P. falciparum* detection using the *Pf*EMP1 primer set consistently outperforms the traditional 18S primer set, detecting as little as 0.01 pg of *Plasmodium* gDNA and 9.3 parasites/mL compared to 0.07 pg and 98.2 parasites/mL for the *Pf*18S primer set. The clinical performance of the *Pf*EMP1 and *Pf*18S gene targets was determined in hospital-based blood film positive clinical malaria samples collected on filter paper and in field acquired blood samples of asymptomatic individuals from Ghana. *Pf*EMP1 amplification consistently outperforms *Pf*18S-based RT-PCR detection, identifying 100% (20/20) of dried blood spot samples and 70% (69/98) of asymptomatic individuals as positive versus 55% (11/20) and 54% (53/98), respectively, for 18S amplification. In conclusion, a *Pf*EMP1-based RT-PCR test has applications in detecting acute filter paper collected and low-grade asymptomatic infections in field who have a potential to convert into severe malaria in children and serve as reservoirs of transmission in both low and high transmission endemic areas.

## Methods

### Primers

Primer oligonucleotides were purchased from Eurofin Genomics (Louisville, Kentucky). The primer sequences employed in this study are: 18S F: CTTAACCTGCTAATTAGCG, 18S R: ATTCCTCGTTCAAGA TTAATAATT; EMP1 F: AAGAAAACGAATTATTTGGGACA, EMP1 R: AGAAACATCAGTATTCAACGTT. SYBR green RT-PCR mastermix was purchased from Bio-Rad (Hercules, CA, USA).

### Human blood samples

*Plasmodium falciparum*-infected blood smear positive samples from patients in Ghana used for this study were collected on Whatman^®^ FTA Elute cards (GE Lifesciences, MA, USA) at Kpone Katamanso District Hospital in the Greater Accra Region of Ghana, a malaria endemic country which recorded about 10 million malaria cases in the year 2015. The study site was developed for multidisciplinary malaria studies by the University of Ghana School of Public Health [13]. Kpone Katamanso district is a peri-urban district located in the coastal-savannah zone that occupies an area of 215.4 km^2^ and has an estimated population of 50,225. While malaria transmission intensity of the region varies seasonally, infections are acquired year-round. Study participants were 1 month to 16 years old children and asymptomatic adults who were screened in the Kpone Katamanso District Hospital.

### Preparation of *Plasmodium falciparum* gDNA

*Plasmodium falciparum* 3D7 parasites were cultured in vitro according to previously published protocols [14] utilizing O^+^ human red blood cells purchased from Interstate Blood Bank (Memphis, TN). Parasitaemia was determined by routine Giemsa staining of blood smears. Final parasitaemia was measured and the number of parasitized red blood cells (pRBCs) per µL was obtained. Isolated ring stage *P. falciparum* parasites from culture were spiked into whole blood and serially diluted. For spiked sample analysis, gDNA was extracted from 1 mL of whole blood at each parasite dilution. Alternately, approximately 100–200 µL of blood was utilized from capillary collection tubes collected from asymptomatic blood smear negative adults from Ghana. For both spiked and Ghanaian blood samples, lysis of RBCs was induced by adding saponin (Sigma, MO, USA) to a final concentration of 0.15%. Remaining cellular material was sedimented by centrifugation at 16,000×*g* for 10 min and washed three times with 1× PBS. gDNA from the pellet was obtained using the QIAmp DNA Blood Mini Kit according to the manufacturer’s instructions and eluted in 60 µL ultrapure water. gDNA extracts from dried blood samples spotted onto Whatman^®^ FTA Elute cards (were prepared according to the manufacturer’s instructions.

### Real-time polymerase chain reaction

Real-time amplification reactions were performed on a QuantStudio 3 RT-PCR instrument (Applied Biosystems, Foster City, CA, USA) using a SYBR Green mastermix from Biorad (CA, USA). The thermocycler protocol was: 95 °C for 5 min; a 40 repeat cycle of 95 °C for 15 s, 57 °C for 30 s, and data collection and elongation at 72 °C for 1 m. Cut-off threshold cycle values were determined for each RT-PCR plate run based upon the mean threshold of included negative (uninfected) control well results minus 1 standard deviation. All results are displayed as signal-to-cut-off ratios using a simple ΔC_t_ method (Fold = 2^(Mean Ct,cutoff − Mean Ct,sample)^.

### Probit analysis

Approximately 40 mL of whole blood was spiked with differing concentrations of *P. falciparum* ring stage parasites (ranging from 1 to 100 parasites/mL). Genomic DNA was isolated from 30 unique 1 mL aliquots drawn from these larger volumes. Extracted genomic DNA from each aliquot was tested by real-time PCR amplification utilizing both the *Pf*18S and *Pf*EMP1 primer sets. The MedCalc statistical software package (MedCalc Software, Belgium) was used to perform probit regression on the RT-PCR data and determine the 95% reactivity threshold and corresponding confidence intervals.

## Results

### Sequence analysis of *PfEMP1* gene amplicon in *P. falciparum* genome

**Fig. 1:**
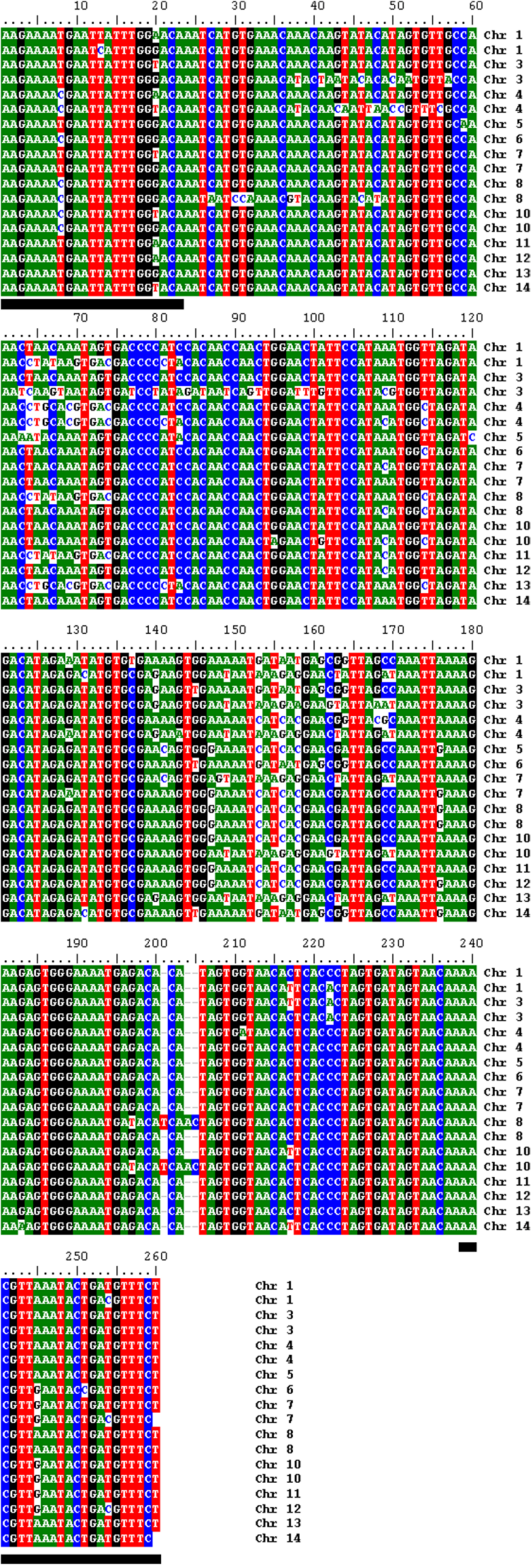
Abundance of *Pf*EMP1-specific amplicon in *Plasmodium falciparum* genome. Primers used in the RT-PCR assay amplify a region of 260 nucleotides located in the cytosolic acidic terminal segment (ATS) region of *Pf*EMP1. Matching conserved sequences in the *P. falciparum* (3D7) genome were identified using an NCBI BLAST search. Chromosomes with homologous sequences are displayed in the figure. The conserved amplification region was found in 18 different loci reflecting significantly higher abundance than the 5 copies of the 18S gene in the *P. falciparum* genome. Black bars represent forward and reverse primer binding positions

To identify a novel and abundant target sequence for RT-PCR-based detection of *P. falciparum* genomic DNA that would potentially outperform standard 18S amplification methods, the sequences of members of the *Pf*EMP1 multi-gene family, which consists of approximately 60 unique variants present per genome, were analysed. Specifically, primers were designed to amplify strongly conserved gene regions that maximize the number of amplification targets. Primer sequences exhibited 91.3–100% identity to a maximum of 18 possible sites of amplification (Fig. 1) within the cytosolic acidic terminal segment of *Pf*EMP1. As this sequence resides on the intracellular side of the erythrocyte, there is substantially less selective pressure for antigenic variation. A maximum of two base-pair mismatches far from the 3′ end of each primer was allowed. Thus, when compared to the 5–8 available genomic copies of *Pf*18S ribosomal subunit present, this *Pf*EMP1-based RT-PCR assay would potentially improve detection capability by offering a more abundant amplification target.

### Limit of detection of *P. falciparum* gDNA

**Fig. 2:**
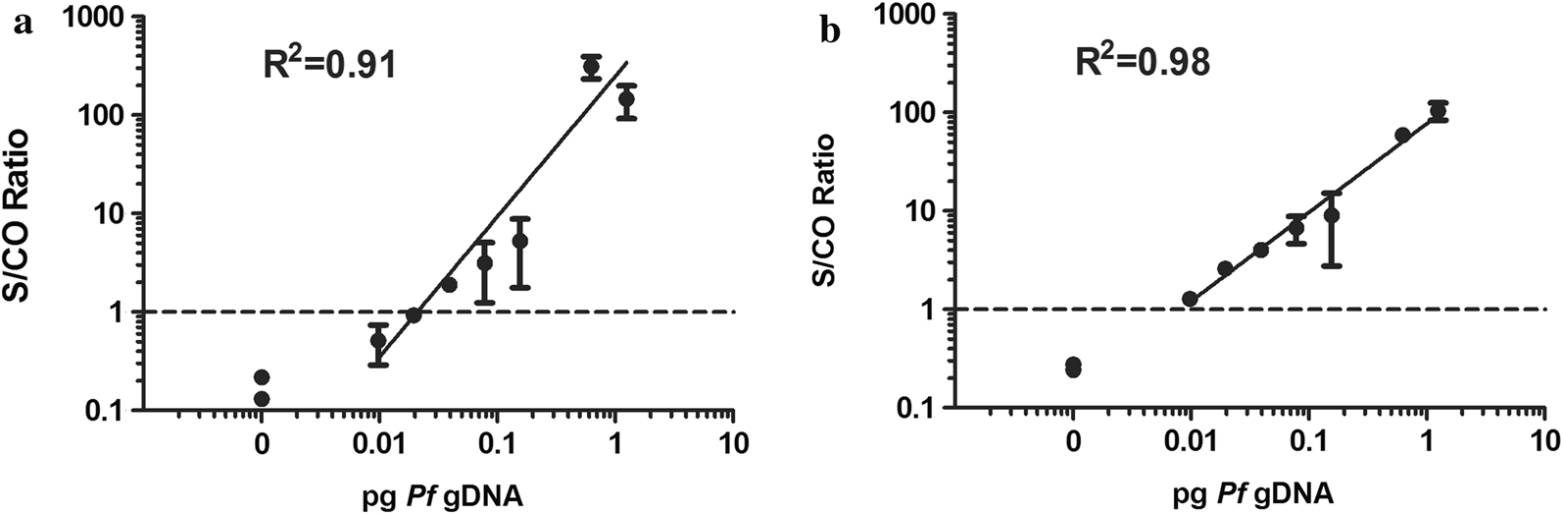
Analytical sensitivity of *Pf*18S and *Pf*EMP1 primers for serially diluted parasite genomic DNA (gDNA). A linear regression identifying the limit of detection for each primer set at the cutoff threshold of the assay. **a** The 18S primers detected as little as 0.07 pg. **b** *Pf*EMP1 primers could detect 0.01 pg of parasite gDNA

To compare the sensitivity of *Pf*18S and *Pf*EMP1 as detection targets, both primers were evaluated in RT-PCR against purified parasite gDNA that was serially diluted in ultrapure water. The *Pf*18S primer set detected as few as 0.07 pg of *Pf* gDNA (Fig. 2a) whereas the *Pf*EMP1 primer set detected as little as 0.01 pg of *Pf* gDNA (Fig. 2b) showing a sevenfold improved sensitivity in detection of *P. falciparum* blood stage parasite gDNA.

### Determination of primer limit of detection (LOD) by probit analysis

**Fig. 3:**
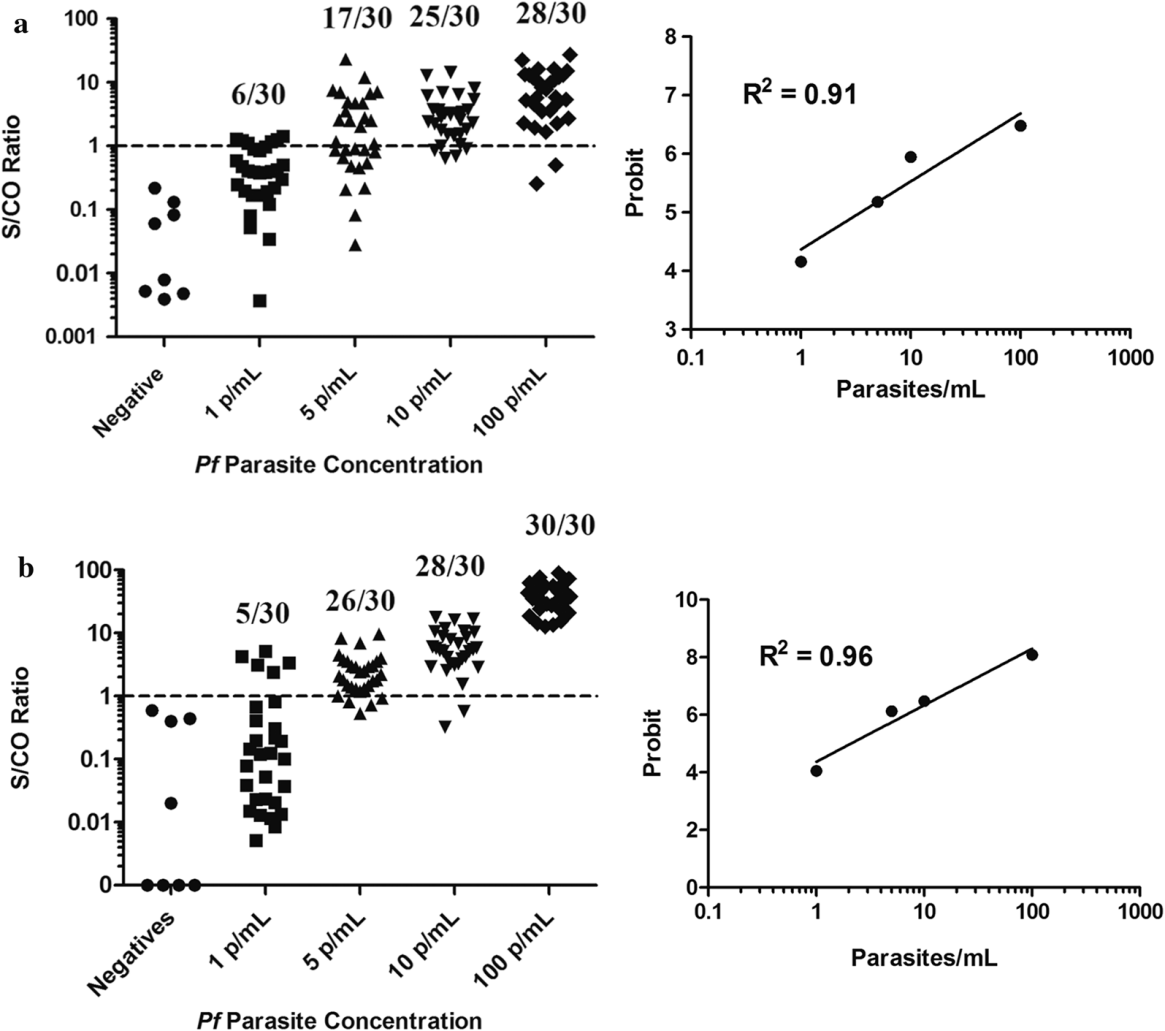
Determination of analytical sensitivity for *Plasmodium falciparum*-infected red blood cells by probit analysis. gDNA extracted from 30 independent 1 mL aliquots taken from the stock parasite dilutions and analysed by RT-PCR using both primer sets. Probit regression was performed using the MedCalc statistical software to identify the 95% reactive threshold. **a** The 18S primer set demonstrated a 95% reactivity of 98.2 parasites/mL (95% CI 64.5–131.9). **b** *Pf*EMP1 primer set demonstrated a 95% threshold of 9.3 parasites/mL (95% CI 6.36–17.8)

Approximately 40 mL of whole blood was spiked with either 100, 10, 5, or 1 parasite/mL of *P. falciparum* blood-stage parasite. gDNA was extracted from 30 unique 1 mL aliquots taken from each stock 40 mL preparation and evaluated in RT-PCR using both *Pf*18S and *Pf*EMP1 primer sets. The MedCalc statistical software package was used to perform probit regression analysis to calculate the parasite concentration at the 95% detection threshold. The probit analysis demonstrates that the 95% detection limit is 98.2 parasites/mL (95% CI 64.5–131.9 parasites/mL) for the *Pf*18S primers (Fig. 3a) and 9.3 parasites/mL (95% CI 6.36–17.8 parasites/mL) for the *Pf* EMP1 primer set (Fig. 3b).

### Detection of *P. falciparum* in dried blood spots and serum samples from Ghana

**Fig. 4:**
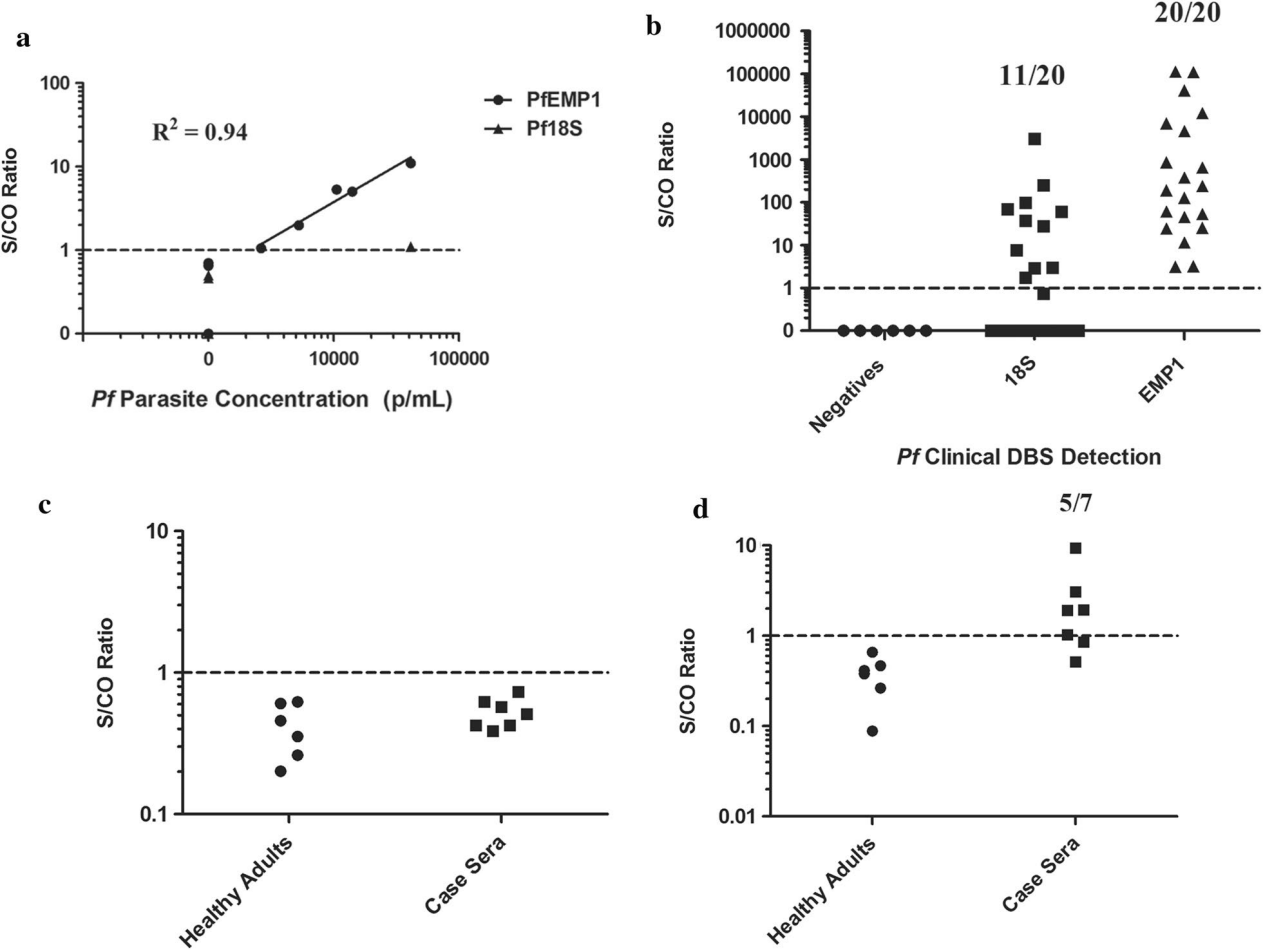
Detection of *Plasmodium falciparum* in dried blood spots from blood smear positive individuals in Ghana. **a** The 18S primer set detected only the highest parasite dilution (41,360 parasites/mL) while the *Pf*EMP1 primer set detected the lowest parasite concentration tested corresponding to 2640 parasites/mL. **b** The 18S primer set identified 55% (11/20) smear positive samples as positive whereas the EMP1 primer set identified 100% (20/20) of smear positive samples as positive. gDNA was extracted from sera demonstrating high reactivity (OD_450_ ≥ 1) against *P. falciparum* circumsporozoite protein measured in ELISA and amplified using both primer sets, **c** *Pf*18S primers failed to amplify from any of the sera samples. **d** *Pf*EMP1 primer set identified 5/7 (71.4%) sera samples as positive for *P*. *falciparum* nucleic acid

To evaluate the clinical sensitivity of each primer set, Ghanaian blood samples that were microscopically positive were assessed for *P. falciparum* reactivity by RT-PCR. Dried blood spots (DBS) employ a small drop of blood on filter paper and are highly utilized in endemic areas due to their relative ease of use and stability at room temperature [15]. Briefly, patient specimens were collected on Whatman FTA-elute filter paper cards in volumes of approximately 10–20 μL and gDNA was isolated according to the manufacturer’s instructions. Importantly, the sensitivity of any pathogen detection assay utilizing dried blood spots is generally lower than whole blood due to the relatively small volume being sampled [16, 17]. To empirically estimate the difference in analytical sensitivity of the RT-PCR assay between gDNA extracted from dried blood spots versus whole blood, gDNA was extracted from both 20 μL of parasite-spiked blood and 20 µL of unspiked, uninfected blood utilized as a negative control that were spotted onto FTA elute cards (Fig. 4a). The *Pf*18S primer set only marginally detected the highest parasite concentration tested (41,360 parasites/mL) while the *Pf*EMP1 primer set successfully reacted to as few as 2640 parasites/mL. The superior sensitivity of *Pf*EMP1 amplification was also confirmed by analysis of dried blood spots collected from individuals in a malaria endemic area of Ghana. The *Pf*18S primer set identified only 11/20 (55%) samples as positive, with 9/20 samples showing no amplification at all (Fig. 4b). In contrast, the *Pf*EMP1 primer set successfully identified 20/20 (100%) blood smear positive samples as positive (Fig. 4b).

Performance of both *Pf*18S and *Pf*EMP1 primer sets for *P. falciparum* detection in serum was also evaluated. Patient serum, as opposed to whole blood samples which are typically assessed in parasite nucleic acid tests, are more commonly stored long-term [18]. Thus, adequate detection of *P. falciparum* from stored serum samples could allow for more extensive retrospective analyses on existing patient-acquired material. These results demonstrated that, while both primer sets showed no amplification in the healthy adult samples, only the EMP1 primer set successfully identified 5/7 (71.4%) sera from infected individuals as positive (Fig. 4c, d).

### Detection of *P. falciparum* in asymptomatic blood smear negative whole blood samples

**Fig. 5:**
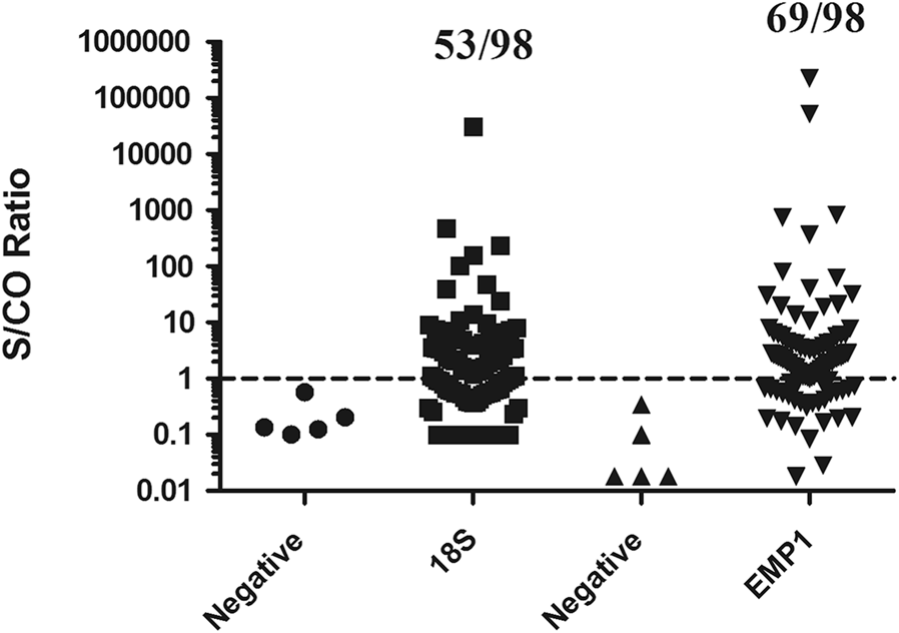
Detection of *Plasmodium falciparum* in asymptomatic blood smear negative individuals from Ghana. gDNA was extracted from whole blood samples collected from children and adults who exhibited no symptoms of malaria and had negative blood smears. *Pf*18S primers identified 53/98 (54.1%) of samples as positive for *P. falciparum* gDNA whereas *Pf*EMP1 primers identified 69/98 (70.4%) blood samples as positive. These differences are statistically significant when evaluated by McNemar’s test and demonstrate *Pf*EMP1 primers possess superior sensitivity for the detection of low burden parasitaemia and more comprehensive identification of infectious reservoir

The clinical performance of *Pf*18S and *Pf*EMP1 primers in detecting submicroscopic asymptomatic infections was evaluated in individuals living in an endemic area of Ghana. Specifically, these blood samples reflect asymptomatic adults who were diagnosed as negative for *P. falciparum* infection by blood film microscopy. *Pf*18S primers identified 53/98 (54.1%) of samples as positive for *P. falciparum* gDNA whereas *Pf*EMP1 primers successfully detected 69/98 (70.4%) (Fig. 5, Table 1). These differences in sensitivity are statistically significant as measured via McNemar’s test (P < 0.01).

**Table 1:** Detection of *Plasmodium falciparum* gDNA in asymptomatic individuals from Ghana

	EMP1		Total
	+	−	
18S			
+	44	9	53
−	25	20	45
Total	69	29	98

## Discussion

Blood-film microscopy is considered the gold-standard for malaria diagnosis. While generally adequate for diagnosis of acute malaria, microscopy lacks the sensitivity to detect low-grade asymptomatic infections as revealed by the application of molecular tests that detect the parasite nucleic acid. Since submicroscopic malaria infections are important sources of further transmission, identification of this population group is critical for accurate estimation of malaria incidence rate in an endemic area. In semi-immune individuals who live with chronic exposure to malaria parasites, asymptomatic low-grade parasitaemia can persist for a year or more [19]. Failure to comprehensively identify these individuals infected with low burden parasitaemias that may not be detected by currently used microscopy, PCR assays, or Rapid Diagnostic Tests, dramatically reduces the effectiveness of Mass Screen and Treat (MSAT) strategies targeting the infectious reservoir of malaria [20, 21].

In this work, the performance of an RT-PCR-based screening assay for *P. falciparum* employing primers that amplify members of the *Pf*EMP1 *var* gene family was characterized. The data establish that *Pf*EMP1 primers exhibit a superior analytical sensitivity of 9.3 parasites/mL in whole blood versus the 98.2 parasites/mL limit of detection for the standard *Pf*18S amplification. Accordingly, amplification of *Pf*EMP1 identified 16% more whole blood samples from asymptomatic adults who were blood film negative as positive than standard *Pf*18S amplification establishing the superior efficacy of this molecule in detecting low grade parasite burdens missed by other methods.

Results from analytical and clinical characterization studies suggest that the *Pf*EMP1-based RT-PCR assay could provide a more comprehensive estimation of the infectious reservoir of malaria and simultaneously allow better targeting of treatment and transmission-blocking interventions. In particular, this assay would reduce false-negative results associated with malaria screening assays of inadequate sensitivity in areas of less intense transmission where chronic parasite burdens are generally low [22]. Additionally, modeling studies have shown that dramatic increases in the sensitivity of malaria diagnostic assays, like that observed with *Pf*EMP1-based amplification, are required to exert any substantial effect on malaria transmission in regions of high intensity. According to one report, application of higher sensitivity assays accelerates the interruption of malaria transmission in an area. Accordingly, deployment of diagnostic assays of superior sensitivity would render overall public health benefits and reduce the organizational and overhead costs of running programmes, such as MSAT campaigns thus remaining a critical component of global malaria eradication efforts [21].

Collection of blood samples as DBS on filter paper is highly amenable for field studies due to ease of sample collection and storage. However, detection of *P. falciparum* from the smaller volumes of blood typically collected as DBS is less sensitive than screening larger volumes of whole blood [16, 17]. Additionally, a recent study has shown that overall PCR reactivity of even high parasite burden DBS declines over time when not stored at − 20 °C [23]. These fundamental sample constraints necessitate a more sensitive diagnostic assay to ensure no loss of parasite detection in valuable field samples acquired in epidemiological or vaccine-related studies. The results demonstrate that *Pf*EMP1-based RT-PCR amplification improved *P. falciparum* parasite detection of microscopically positive Ghanaian blood samples as DBS when compared to standard *Pf*18S amplification methods. Specifically, *Pf*EMP1-mediated gene amplification demonstrated a sensitivity of 2460 p/mL versus a limit of detection of at least 41,360 parasites/mL for 18S-based detection. Additionally, *Pf*EMP1 primers successfully amplified 100% of blood smear positive individuals from dried blood spot samples whereas *Pf*18S amplification only detected 11/20 (55%) DBS samples as positive. Importantly, the failure of *Pf*18S amplification to consistently match results from gold standard blood smear microscopy of clinical samples collected as DBS indicates a limitation in this blood collection method and inherent challenges in conducting large field studies in endemic areas. Cumulatively, these data demonstrate that RT-PCR amplification of *Pf*EMP1 provides a superior method of parasite detection among DBS spots collected in field sites and ensures that any potential losses of sensitivity are minimized when this widely used sample format is employed.

The analytical and clinical performance studies establish *Pf*EMP1 as a superior biomarker for RT-PCR-based detection of both submicroscopic infections in samples collected as whole blood and patent parasitaemias from samples collected as DBS. These results support the identification of potentially novel abundant diagnostic markers as a means of improving the sensitivity of pathogen gDNA detection. While *Pf*EMP1 is a well- known and characterized molecule associated with malaria pathogenesis [24–26], no reports have thus far attempted to harness this high copy multi-gene family as an amplification target in nucleic acid-based screening assays for malaria. All *Pf*EMP1 variants are high-molecular weight parasite proteins (200–450 kDa) inserted into the membranes of *P. falciparum*-infected red blood cells. *Pf*EMP1 is the major constituent protein of knobs that are responsible for sequestration of infected RBCs in the peripheral vasculature that allows immune evasion of later-stage parasites [27, 28]. The conserved cytosolic portion of *Pf*EMP1 is anchored to the RBC cytoskeleton by lysine-rich membrane associated PHISTb (LyMP) [29, 30]. Although 60 variants of *Pf*EMP1 exist, only a single *Pf*EMP1 is expressed during any given 48-h cycle. All other unexpressed versions of parasite *Pf*EMP1 are silenced by multiple different mechanisms such as histone modifications [31], promoter-intron ‘pairing’ [32], and tethering of the *var* genes at the nuclear periphery [33]. Thus, while *Pf*EMP1 may not serve as a superior transcript for RNA-based amplification assays, the high number of gene copies of *Pf*EMP1 in the malaria genome makes it an ideal candidate diagnostic marker for the detection of parasite DNA in blood. In this work, primers were designed to amplify a strongly conserved gene sequence encoding the cytosolic acidic terminal segment (ATS) region of *Pf*EMP1. The finalized forward and reverse primers possess at least 91% identity to 18 different loci within the *P. falciparum* genome which offer more abundant targets of amplification than the available 5–8 genomic copies of the 18S ribosomal subunit gene. While the extracellular portion of *Pf*EMP1 is subject to immense selective pressure to evade the immune system, the cytosolic domain amplified in the assay and located in the second exon of the gene is significantly less diverse [34, 35].

## Conclusion

Apart from developing countries, malaria also presents a challenge in industrialized countries where autochthonous transmission does not occur. In these countries, malaria presents a significant public health challenge through imported clinical cases and as transfusion transmitted malaria [4, 5]. Global malaria surveillance is a priority of the World Health Organization, however, reported cases are often incomplete, lacking confirmatory testing, and many cases are unreported because of misdiagnosis. Most incidences of imported or transfusion-related cases in nonendemic countries can be traced to prior residents of malaria endemic areas [36]. Due to acquired immunity, these individuals would be potentially asymptomatic and maintain an infectious reservoir characterized by low parasite burden. Assays of higher sensitivity would be required to identify prospective at-risk blood donors who may have travelled to or were prior residents of malaria endemic areas. In summary, this work established an RT-PCR-based assay for the detection of parasite gDNA that relies on amplification of *Pf*EMP1 gene sequences. The use of *Pf*EMP1 as a diagnostic marker of malaria infection has demonstrated superior analytical and clinical sensitivity when compared to the current laboratory standard of 18S RT-PCR amplification; therefore, the proposed assay ensures the detection of even asymptomatic, low parasite burden individuals that present the greatest risk to the blood supply.
